# Affordances of School Ground Environments for Physical Activity: A Case Study on 10- and 12-Year-Old Children in a Norwegian Primary School

**DOI:** 10.3389/fpubh.2022.773323

**Published:** 2022-04-01

**Authors:** Lise Kjønniksen, Nora Wiium, Ingunn Fjørtoft

**Affiliations:** ^1^Faculty of Humanities, Sports and Educational Science, University of South-Eastern Norway (USN), Kongsberg, Norway; ^2^Faculty of Psychology, University of Bergen, Bergen, Norway

**Keywords:** school children, physical activity, accelerometer, school ground environments, affordances

## Abstract

Several studies have focused on how different school ground environments can stimulate physical activity (PA) in children. This study aimed to investigate the contributions of two school ground environments (a constructed schoolyard and a natural forest) in moderate to vigorous physical activity (MVPA) of Norwegian school children in the 5th and 7th grades. This study described two school ground environments that provided large and multifunctional spaces, giving the children several affordances for being physically active during the school day. The constructed schoolyard afforded a space of 44 m^2^ per child and had an access to sports and game courts and various types of equipment for PA. The natural forest provided a space of 50.6 m^2^ per child and had a varied landscape for activities that afforded a wide range of PA. On average, the children engaged in 50% of the 60-min period of MVPA when playing in the natural and constructed play settings. The two different environments, thus, contributed equally to the daily MVPA of the school children. The findings can inform policies and programs aiming at promoting recommended levels of PA among children using school outdoor environments that may eventually have implications for the physical and mental health of school children during the current pandemic.

## Introduction

The positive effects of physical activity (PA) on children's health have been highlighted in many scientific studies (1–3) as well as international documents (4, 5). To promote a healthy lifestyle among school children, several countries, such as Norway, have adopted the global recommendation of at least 60 min of moderate to vigorous physical activity (MVPA) every day for children and youth.

Drawing on a population survey using accelerometers across Norway, the Norwegian Directorate of Health (6) reported that in 9-year-olds, 64% of girls and 81% of boys met the global recommendation. Among 15-year-olds, equivalent proportions were 40 and 51%. The level of PA was found to decline by age. Gender differences existed in all age groups, showing that boys were more active than girls.

As attending school is compulsory for most children, especially in Western countries, schools have an important role in promoting PA throughout the school day. Children spend most of their daytime at school, and, consequently, school grounds are important arenas that can be used to facilitate PA in children (7). Bell and Dyment (8) found that school-aged children spend ~25% of their school day on school grounds, making schools important sites to engage children at healthy levels of physical activity. A recent study on Nordic-Baltic schoolyards found a gap between the design of schoolyards and school childern's preferences (9), indicating the need for more knowledge of children's needs and preferences of school ground affordances.

To ensure that school-aged children meet the recommended level of PA, the Norwegian Ministry of Education introduced the Amendment to the Act of Education in 2009 (10). In this Amendment, schools were instructed to provide 60 min of extracurricular PA a week for school children in grades 5–7 (10, 11, and 12 years old), in addition to existing physical education lessons. Thus, schools were supposed to provide more physically active and varied school days to meet the global recommendation of daily PA. Several Norwegian schools use their school ground environments to implement the Amendment. A national evaluation report indicated that these regulations are somehow difficult to implement (11). The evaluation also demanded more reports and research on the implementation of the mandate and its success in practice. Indeed, there is evidence from other studies that characteristics of the physical environment around schools may influence children's PA levels (12, 13).

In Norwegian schools, the tradition is to be outdoors using their school grounds for PA both during recess and in physical education classes. In fact, being outdoors is part of the Norwegian culture and a natural as well as integrated part of the school day. Norwegian guidelines (14) recommend an outdoor space of 30 m^2^ per child in relation to the number of students in a school. Facilities of a school ground should be varied and customized to different age groups and functions. The physical outdoor environment around a school may vary according to geography and location, rural, or urban (14).

Earlier research suggests that children accumulate more PAs when playing outdoors than indoors, as outdoor environments promote more PAs than indoor environments (15, 16). For example, Cooper et al. (17) found that the intensity of PA was significantly higher outdoors than indoors. It is likely that environmental factors may influence PA levels in children, although these are dependent on the facilities provided (18–20). The authors observed that infrastructures, such as buildings, roads, and pavements, were used for light activities, and that green environments, such as gardens, parks, grassland, and farmland, were supportive of vigorous activities. Furthermore, Mårtensson et al. (21) found that settings with a mixture of green and built environments were favorite playgrounds during recess in 10–13 year-olds. Their findings are in line with those of (22) who found that school physical environment was the most effective means of enhancing PA in children. Thus, school ground environments may afford opportunities for PA, and the contextual diversity of schoolyards and natural environments appear to be crucial for promoting PA (18). An observational study on children's behaviors across two playgrounds found that fixed equipment and open play spaces encourage various levels of play and physical activity (23). Despite the contribution of these earlier studies, there is a knowledge gap regarding affordances of school ground environments for children's physical activity.

The physical environment of school grounds refers to objects and structures that turn landscapes into learning arenas and tasks that are stimulating, challenging, explorative, and diverse (24). Gibson's theory of affordances (25) explains how a physical environment can provide a context for human behavior and learning. Physical environments stimulate different behaviors and offer usage possibilities that are linked to affordances of a specific environment. Affordances of an environment can be potential and/or actualized (25, 26). Potential affordances refer to all possibilities that an environment offers, e.g., rocks can afford climbing, and an open field may afford running and jumping. Actualized affordances are the context between a physical environment and a child, which is expressed by the response of the child and visualized through specific types of physical activity, which may reflect different intensity levels of respective activities promoted by different affordances of environments. This response can be observed or measured as the activity of a child. Heft (27) defined affordances as functional characteristics of environmental features that are significant for an individual, introducing concepts of environmental features to be usable, like something that fits the hand becomes “throw-able”, a tree or a rock being “climb-able”, and an open space being “run-able”. Lerstrup and van den Bosch (28) have used the taxonomy of Heft in describing how pre-school children are using traditional outdoor playground contra a forest, indicating the importance of the user-environment activity relationship.

In this study, potential affordances are possibilities in environments that may offer physical activity to school children. Our main aim was to map potential affordances for physical activity in two school environments as well as monitor the level of physical activity in 10- and 12-year-old school children in the two different environments during a 60-min extracurricular PA a week provided to these age groups (10).

The following research questions were examined:

1. What are the potential affordances for PA in the two school ground environments, the constructed schoolyard and the natural forest?
2. How do the two school ground environments afford MVPA in schoolchildren?
3. How do the two school ground environments afford MVPA across grades and gender?

With these research questions, we explored how the 60-min extracurricular PA a week (the National Amendment) met the national and global recommendations for daily PA in school children.

## Materials and Methods

### Study Design

This was a descriptive case study with a quasi-experimental design (29), and it included two groups (school-aged children in the 5th and 7th grades) but had no control group. The independent variables are the constructed schoolyard and the natural forest together with their respective affordances for PA. The dependent variable is the PA levels in children in the two school ground environments measured with accelerometers.

### Case Selection and Participants

A primary school located in a rural area of south-eastern Norway with a diverse and multifunctional school ground including a natural forest was selected as the case for this study. The selected primary school had a total of 200 pupils in grades 1–7 (6–12 years). School children in grade 5, ~10 years old (*n* = 27, 16 boys and 11 girls) and grade 7, ~12 years old (*n* = 28, 15 boys and 13 girls) were selected as participants for the study. To accommodate the Amendment to the Act of Education (10), these two school classes were each assigned by the school authorities to have 60 min of extracurricular PA a week during a school year.

### Collection of Data

All school children in grades 5 and 7 in the case study school were eligible to participate. Children's participation in this study was voluntary and in accordance with the Declaration of Helsinki. Written consent was obtained from the school and parents prior to the study. All monitoring, collection of data, and analysis were treated anonymously and in line with ethical guidelines. The project was approved by the NSD-Norwegian Centre for Research Data. The collection of data included mapping and describing the two school ground environments and facilities for PA. The PA of the experimental groups was objectively monitored using accelerometers during the 60-min period in the two school ground environments.

### Mapping and Describing Potential Affordances of School Ground Environments

The school was located in a rural area of an agricultural district. The school ground constituted a constructed schoolyard and a natural forest, and was mapped using a standard registration form for field observations to identify school ground areas, facilities, and landscape characteristics around the school area as well as potential affordances for PA (30). Ortophoto maps (Google maps: Norway in pictures, 2010; https://maps.google.no/maps) were applied as a basic source for visualizing the school ground and elaborated further into a topographic map (Norgeskart.no) visualizing environmental qualities of the two schoolyard landscapes. Mapping results were processed with illustrator tools using the program Adobe Illustrator CC. The two school ground environments were mapped and described by the authors, indicating landscape qualities and facilities that potentially afforded PA in the children.

### Assessment of Physical Activity

We assessed the PA of the school children in accordance with the global and national recommendations for PA in children and adolescents. These recommendations require at least an average of 60 min per day of MVPA of mostly aerobic physical activity (5, 31), and are according to the guidelines from Utdanningsdirektoratet (10). The PA of 5th- and 7th-grade children was monitored for 60 min in the schoolyard and 60 min in the natural forest on two different school days. Each grade was measured once in each environment. In the two environments, the children could freely engage in different activities without the direction of teachers. The study was conducted in early autumn with a mean temperature of 10–12°C and good weather conditions (not raining).

### Monitoring of Physical Activity

Accelerometers, *ActiGraph GT1M* (Actigraph, LLC, Fort Walton Beach, FL, United States) were used to monitor PA. The ActiGraph GT1M is a sturdy and compact dual-axis accelerometer that measures and records steps during vertical activities. The pupils were instructed to fix the accelerometer in the right hip position. Monitoring time of the PA was 60 min for each grade in each of the school ground environments. An epoch period of 10 s was selected for monitoring PA, which corresponded with earlier Norwegian surveys on PA in children and youth (6). The instrument measures vertical movements related to duration, intensity, frequency, and variation over time (Actigraph LLC, Pensacola, FL, U.S.). The cutoff point for MVPA was defined as 2,000 counts per minute. This cutoff point has been used in previous studies to define MVPA (32–34) and is comparable to the age-specific cutoff for 8.5-year-old children (35). The cutoff point at 2,000 counts per minute has been applied in previous Norwegian surveys on PA in children and youth (6).

### Analysis of Data

Three school children out of the initial 55 participants in the study dropped out. The analysis of data, therefore, included PA levels from 52 school children. Descriptive analyses were conducted for PA levels in the constructed schoolyard and the natural forest across grades and gender. A series of independent sample *t*-test analyses was conducted to determine differences in MVPA levels on the two school ground environments across gender and grade. In addition, paired sample *t*-tests were carried out to assess differences in gender and grade with respect to MVPA levels in the two school ground environments. A two-way ANOVA was conducted to examine the main and interactive effect of grade and gender on PA levels in the two school ground environments. All the analyses were conducted using the SPSS statistical program.

## Results

### Affordances for Physical Activity in the School Ground Environments

**Figure 1:**
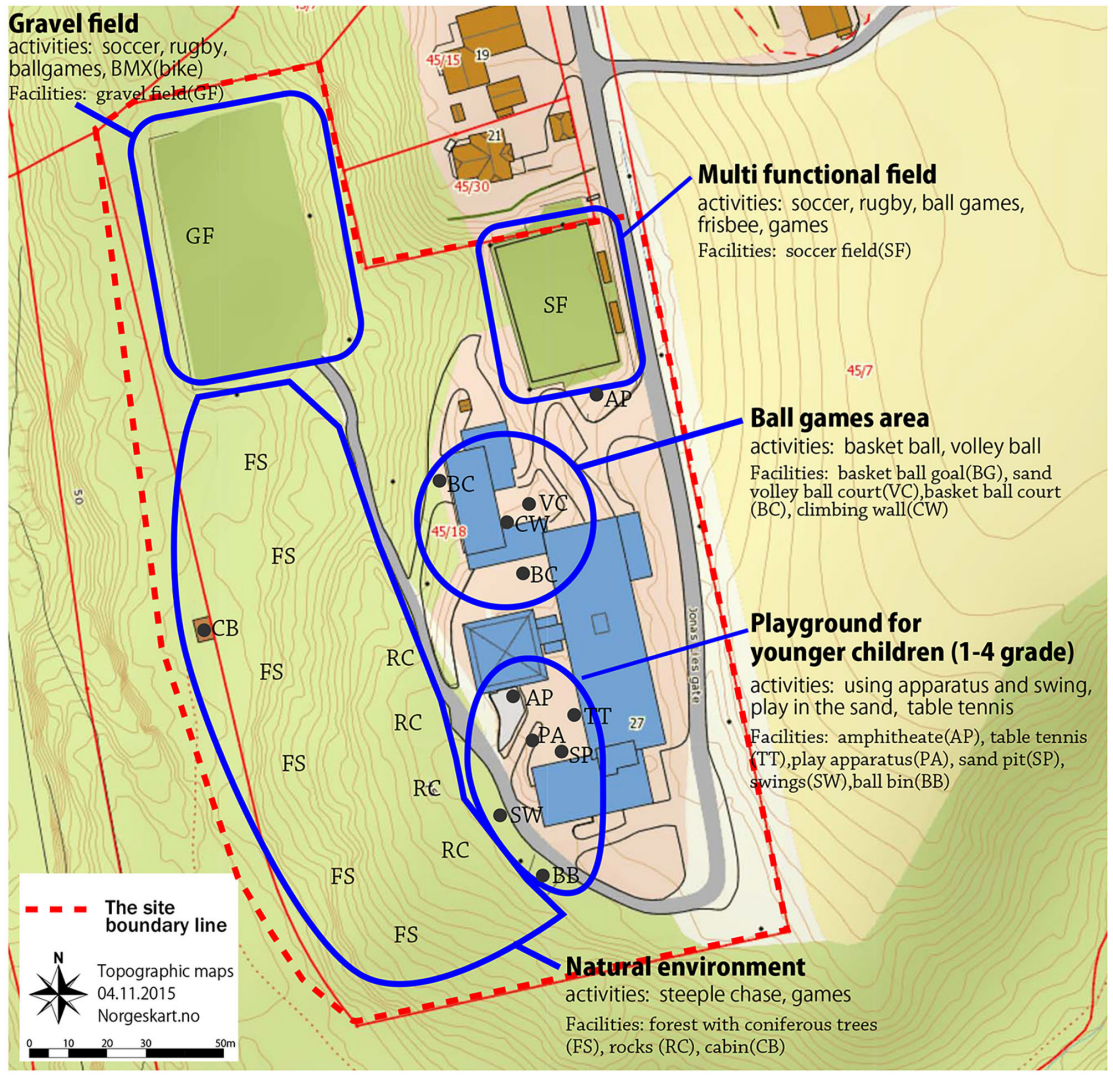
School ground environments including the constructed school yard (multifunctional field, ball game areas, playground for younger children (grades 1–4), gravel field and natural environment consisting of a natural forest (FS), rocks (RC), and cabin (CB).

**Figure 2:**
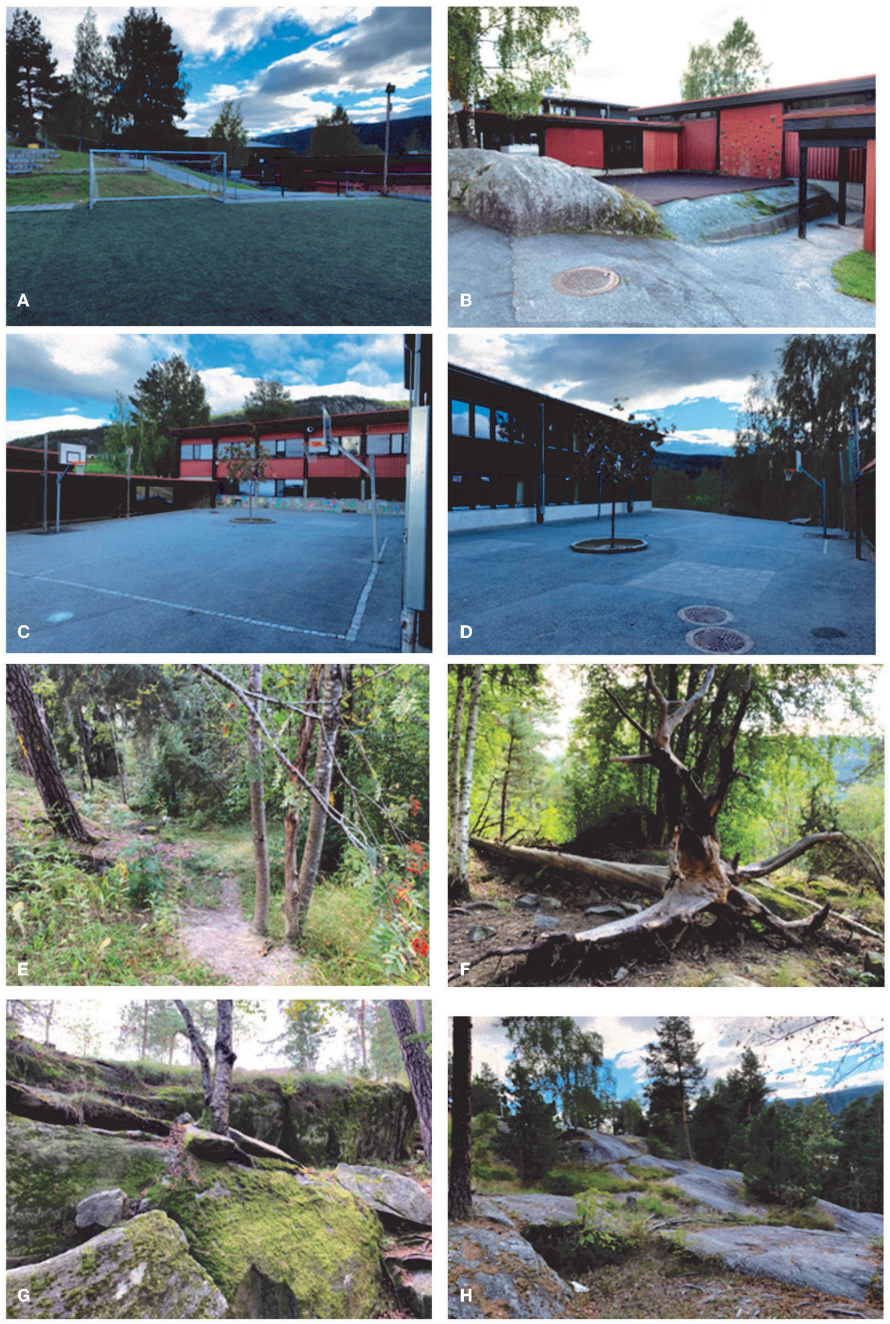
School ground environments showing potential affordances for different activities: The constructed schoolyard environments: **(A)** the multifunctional ballgame court, **(B)** the climbing wall with a soft cover substrate, **(C)** the basketball court, **(D)** the volleyball/ball game court. Environmental affordances of the natural forest: **(E)** cross-country running and hide-and-seek; **(F)** jungle gym, climbing, and balancing; **(G)** climbing and bouldering; **(H)** cross-country running (high speed).

The two school ground environments constituted a total area of 19,007 m^2^, providing an area of 95 m^2^ per child. Thus, in comparison to the recommended school space of 30 m^2^ per child (14), the total school ground was assessed by the authors as large, varied, and multifunctional with many and diverse affordances for PA. The constructed schoolyard (8,874 m^2^; 44 m^2^ per child) constituted a multifunctional field affording traditional games and ballgames. Loose materials, such as balls, skipping ropes, space hoppers, Frisbees, and badminton equipment were also available on the multifunctional field (Figures 1, 2A). Sports and ball game courts for basketball, volleyball, handball, and soccer were available (Figures 1, BC, VC, SF, 2C,D). In addition, there was a climbing wall in one of the school buildings (Figures 1, CW, 2B). All these facilities potentially afforded versatile PAs, such as running, jumping, climbing, throwing, sliding, and biking and were available to the children during the extra 60 min of PA.

The natural forest, which consisted of a mixed coniferous forest, had an area of 10,133 m^2^ (50.6 m^2^ per child) and varied topography including hills, slopes, and rocks, and a mixed vegetation of trees, bushes, heather, and grass (Figure 1 FS, RC). The forest had potential affordances for PAs, such as steeplechase and jungle gym, hide and seek, and different kinds of traditional games. Opportunities for climbing trees and rocks afforded a wide range of body movements (Figures 1, FS, RC, 2E,F). Small paths indicated affordances for running, bushes for hiding, loose materials for constructions, e.g., small cabins, as well as cones and sticks that afforded throwing (Figures 2E–G). All these affordances were available to the children during the extra 60 min of free play.

The gravel field (Figure 1 GF) was located next to the forest and was mainly used for activities, such as soccer, rugby, traditional games, and BMX/bikes. The cabin (CB in Figure 1) located in the forest functioned as a classroom for outdoor learning.

### How School Ground Environments Afforded MVPA in School Children

In the results (not presented in Tables), PA monitoring with accelerometers showed that within a period of 60 min, about half of the participants in the total sample spent 30 min or more in MVPA in each of the two school ground environments: 51.9% in the natural forest vs. 51.1% in the constructed schoolyard. Thus, half of the children were almost equally active in each of the two school ground environments.

### Environmental Affordances of MVPA Across Grades and Gender

Results from the frequency analysis (not presented in tables) showed that mean time spent in MVPA among the school children ranged from 7.4 to 39.2 min in a period of 60 min playtime in the natural forest and from 5.4 to 54 min in a similar playtime period in the constructed schoolyard. Thus, PA in the constructed schoolyard promoted the maximum time (54 min) in MVPA, which was registered by a 5th-grade boy. In Table 1, independent t-test only revealed gender differences in the schoolyard. The boys in in fifth grade spent more time in MVPA (a mean time of 39,18 min) compared to the girls (mean time 23,22 min). Paired sample *t*-test indicated within-gender differences in the 5th grade, where the boys spent more time in MVPA in the schoolyard (a mean time of 39.18 min) relative to their time spent in MVPA in the natural forest (a mean time of 31.63 min), although the significant level was only marginal. The opposite was true for the 5th grade girls, where a lower mean time of 23.22 min in MVPA in the constructed schoolyard was registered compared to their mean time of 31.52 min in the natural forest. There were no within or in-between gender differences in MVPA mean scores in the 7th grade concerning the two school ground environments (Table 1).

**Table 1:** Time spent (in min) in moderate-to-vigorous physical activity (MVPA) among school children in the natural forest and constructed schoolyard: a series of *t*-test analyses.

**Grade**	**Gender**						***t***	***df***	***sig***.
	**Boys**			**Girls**					
	***M***	***SD***	***n***	***M***	***SD***	***n***			
**5th**									
Natural forest	30.70	7.90	16	31.52	6.00	9	−0.27	23	0.516
Schoolyard	39.18	8.88	15	23.22	5.30	9	4.87	22	**0.001**
**7th**									
Natural forest	28.94	7.61	15	26.54	4.39	12	0.97	25	0.542
Schoolyard	28.51	10.62	12	27.13	11.36	11	0.30	21	0.954
**Gender**	**Grade**						***t***	***df***	***sig***.
	**5th**			**7th**			
	***M***	***SD***	***n***	***M***	***SD***	***n***		
**Boys**									
Natural forest	30.70	7.90	16	28.94	7.61	15	0.63	29	0.531
Schoolyard	39.18	8.88	15	28.51	10.62	12	2.85	25	0.548
**Girls**									
Natural forest	31.52	6.00	9	26.54	4.39	12	2.20	19	0.326
Schoolyard	23.22	5.30	9	27.13	11.36	11	−0.95	18	0.098
**Grade**	**School grounds**						***t***	***df***	***sig***.
	**Natural forest**			**Schoolyard**			
	***M***	***SD***	***n***	***M***	***SD***	***n***			
**5th**									
Boys	31.63	7.20	15	39.18	8.88	15	−1.99	14	0.066
Girls	31.52	6.00	9	23.22	5.30	9	2.45	8	**0.040**
**7th**									
Boys	30.58	5.32	12	28.51	10.62	12	0.68	11	0.513
Girls	26.77	4.66	10	25.97	11.27	10	0.22	9	0.833

*M, mean; SD, standard deviation. Bold and italic values highlights the significant differences of results*.

As shown in Table 2, a two-way ANOVA is performed to assess the main and interaction effects of grade and gender on time spent in MVPA in the constructed schoolyard and the natural forest. The results showed a significant main effect of gender: *F*_(1, 43)_ = 9.51, *p* = 0.004, indicating a significant difference between the boys (*M* = 34.43, *SD* = 10.93) and the girls (*M* = 25.37, *SD* = 9.15) and an interaction effect between grade and gender (*p* = 0.013) on time spent in MVPA in the schoolyard. Specifically, the boys spent more time in MVPA in the schoolyard, while the interaction effect confirmed that this observation was mainly among boys in the 5th grade. The explained variance of the model for time spent in MVPA in the schoolyard was about 27%. There were no significant main or interaction effects of grade and gender on time spent in MVPA in the natural forest (Table 2).

**Table 2:** Time spent in MVPA in the natural forest and constructed schoolyard across grade and gender: a two-way analysis of variance.

**Source**	**Type III Sum of Squares**	***df***	**Mean square**	***F***	**Sig**.
***Time spent in MVPA in natural forest***					
Grade	140.40	1	140.40	3.00	0.090
Gender	7.68	1	7.68	0.16	0.687
Interaction (Grade * Gender)	32.05	1	32.05	0.68	0.412
Error	2,246.60	48	46.80		
Total	47,285.67	52			
Corrected Total	2,415.36	51			
R squared = 0.07 (adjusted R squared = 0.012)					
***Time spent in MVPA in constructed schoolyard***					
Grade	130.70	1	130.70	1.45	0.235
Gender	854.03	1	854.03	9.51	***0.004***
Interaction (Grade * Gender)	603.60	1	603.60	6.72	***0.013***
Error	3,860.91	43	89.79		
Total	48,587.92	47			
Corrected Total	5,640.18	46			
R squared = 0.315 (adjusted R squared = 0.268)					

*Bold and italic values highlights the significant differences of results*.

### Summary of Results

This study showed how two school ground environments stimulated PA in school children. The total space of the school ground of 19,007 m^2^ provided a total area of 95 m^2^ per child, of which 44 m^2^ constituted the constructed schoolyard and 50.6 m^2^ the natural forest. Space per child was even larger when only one class of 12–16 children was outdoors at a time. Affordances in the constructed schoolyard and the natural forest supported almost equally the MPA levels in 10- and 12- year-old school children. On average, children engaged in ~50% of the 60-min period of moderate- to vigorous-intensity PA when playing in the natural and constructed play settings. Differences in activity levels were observed between the boys and the girls in grade 5 but not in grade 7.

Despite environmental differences between the two school ground environments, a multifunctional constructed schoolyard and a natural forest, both environments appeared to afford high levels of PA. Generally, the results showed little to no differences in PA in the two school ground environments across grade and gender in relation to time spent in MVPA. Specifically, boys in the 5th grade spent more time in MVPA in the constructed schoolyard than other school children in the sample.

## Discussion

Potential affordances for PA in the two school ground environments were seen as multifunctional with various much space-affording options for PAs. The results showed that both the natural forest and the constructed schoolyard generated MVPA in the schoolchildren. With a total area of 19,007 m^2^, the school ground environments provided an area of almost 51 m^2^ per child in the natural forest and an area of 44 m^2^ per child in the constructed schoolyard for PA, exceeding the Norwegian guidelines for an outdoor space of 30 m^2^ per child in schools with a maximum 450 pupils (14). Space has been discussed in previous studies and appears to be a crucial factor for affordances of PA, especially for meeting the national and global recommendations for daily PA (18). School ground space has been documented in different Nordic and Baltic countries with different recommendations and regulations for space and design of schoolyards (9). As space is important for physical activity at high intensity, this should be important for future studies and designs of schoolyards.

The results showed that a variety of potential affordances in the two environments was associated with healthy levels of PA among the schoolchildren. Even though the actualized affordances of the two environments were not documented, the monitored levels of MVPA in the children in the two environments reflected positive environmental contexts for intensive PA in the two school grounds (see Figure 2). The constructed schoolyard constituted several open areas with asphalt, gravel, and artificial lawns, which were related to PA with high intensity (Figures 2A,C,D). Other facilities like the climbing wall, rocks, and rubber surface afforded varied PAs (Figure 2B). The natural forest afforded running and steeple chase in a rough terrain and different opportunities for PA (Figures 2E–H). Earlier studies have also documented the importance of green environments for play and PA (36–38). Morton et al. (39) conducted a review on the current evidence of school-based PA and physical environment using ninety-three studies on mixed methodological quality. Their findings showed that availability of sufficient space and facilities were considered important for high levels of PA. These findings are in line with our study documenting the importance of space and diversity in school ground environments.

Our findings are consistent with earlier studies on the role of green space and multifunctional school grounds in stimulating PA. For example, Bell and Dyment (8) examined the information provided by parents, teachers, and administrators across several elementary schools in Canada and found that green school grounds appeal to children's interests and support a wide variety of play opportunities that promoted PA. Furthermore, 20 observed that green environments supported vigorous PA, and that boulders, trees, and bushes appeared to encourage moderate PA. A recent study by 40 explored how secondary school students experienced and used school grounds of varying sizes, contents, and designs in PA. Their results indicated that large surface areas and varied contents with ball court, greenery, and multifunctional equipment were valued by students. Thus, creating more “activity-friendly” environments holds a promise for improving PA in school children (40) and students (41).

How the school ground environments afforded MVPA in the school children was monitored with accelerometers. The results showed that within the period of 60 min, about half of the participants in the total sample spent 30 min or more in MVPA in each of the two environments. This is a fundamental contribution to reaching the recommended 60 min of MVPA every day. Consistent with Bell and Dyment (8), the children spend a quarter of their school day in the schoolground. Consequently, school grounds are promising sites that can enable children to meet the recommended 60-min daily PA. Our study showed that access to adequate space and facilities is important for affording PA in school children, a finding that was also documented by (42). School outdoor environments, therefore, should be varied and multifunctional to afford many possibilities for PA among all children.

A main effect of gender and an interaction between grade and gender were found in time spent in MVPA in the constructed schoolyard but not in the natural forest. The findings showed that the boys, particularly those in the 5th grade, were more physically active in the constructed schoolyard than the other school children in our sample. Our findings in the constructed schoolyard are somewhat in line with earlier research studies that have also shown a decline in PA levels with age as well as gender differences, where boys were more active than girls (6, 43, 44). However, there were no gender differences in MVPA in the natural forest, while within-gender differences revealed that girls in the 5th grade were more active at MVPA levels in the forest than in the schoolyard. This may indicate that the forest stimulated more actualized activities for the 5th grade girls than the constructed schoolyard. This needs to be better investigated in future studies with a larger sample along with comprehensive and systematic observations.

Our study on 60 min of extracurricular PA in 5- and 7th-grade school children confirmed that the environmental context for PA supports the national and global guidelines for daily PA in children (5, 31) and confirmed the purpose of the Amendment Act for PA in schools (10). Mainly focusing on the intensity of PA during the 60-min period, the results indicated that the level of recommended daily PA was successfully reached by 50% of the extracurricular time used for PA at intensity levels of MVPA. The finding is in line with a national study on PA in 9- and 15-year-old children (6).

An evaluation report of the Amendment Act of 60 minutes extracurricular PA a week, found no effects of the implementation of the mandate in the evaluated studies (11). Consequently, this study may provide relevant information on the positive effects of the Amendment Act, with focus on the context of supporting school environments.

## Strengths and Limitations

Mapping the characteristics and qualities of school ground environments and examining differences in activity levels in the environments may contribute to the understanding of environmental contexts for promoting PA in school children. The use of accelerometry to quantify activity in the context of environmental affordances is a new methodological approach. This made it possible to describe contextual relations to the levels of PA during the extra 60 minutes a week provided for 5 to 7 graders (10).

The limitations include focus on one school, only examining boys and girls in two grades, and assessing activity levels once in each setting. Our study was limited to two groups, the 5th and 7th grades, and only one class in each grade was included in the study. Thus, our sample was small and may not be representative enough to make any generalized conclusion on PA and time spent in MVPA during the provided extra 60 min among Norwegian school children in grades 5–7, although our findings provide some indication of average level of activity. To increase the reliability and validity of future studies, more schools and school children in appropriate grades should be included in samples. In addition, applying accelerometers solely and achievement of MVPA as a measure for PA might be limitations, as they do not differentiate among qualities of movement patterns including climbing, coordination, and balance, which may not contribute to MVPA levels. Observations could have been conducted as additional methods for explaining how actualized environmental affordances promoted children's versatile movement patterns.

Furthermore, the study was carried out in only one season of the year, which was during early autumn when temperatures were mild and weather conditions were good. It is, therefore, not clear whether school children would be equally physically active if weather conditions were different or temperatures were harsher. As there are four seasons in Norway with unique climate conditions, the study will need to be replicated in all seasons to verify that the school ground environments indeed afford MVPA in school children independent of the season.

## Implications and Conclusion

Despite the limitations of this study, our findings revealed the importance of spacious and varied outdoor school ground environments in children's daily PA. Our findings indicated that diversity of outdoor environments is an important stimulator of daily PA in school children. There are only a few studies in this field, and the expectation is that our study will pave the way for future studies on affordances of school grounds and their relevance for PA in school children. To our knowledge, there are no other Norwegian studies that have described and validated the implementation and effects of the national amendment of extracurricular time for physical activity in 5- to 7th-grade school children. Our study showed that both the natural forest and the multifunctional constructed schoolyard that ensure acceptable play space are important for PA in the school children. This means that school ground environments can have a significant impact on children's PA and can effectively enable MVPA at recommended daily levels. The aspect of affordances should be more emphasized in future planning and renovation of school grounds, as different qualities of school ground landscapes and facilities may inspire children to have increased PA. The quality of school ground environments and impact on PA in children should have wider attention, and hopefully, this study may stimulate more studies in the field. Applying environmental mapping techniques and analyses of potential and actualized affordances for designing multifunctional schoolyards should be future methods in such processes. However, more research is needed to assess the quality and design of school grounds in terms of their affordances for PA, which can eventually help inform effective PA policy and practice. Accordingly, research evidence will expectantly influence policy-makers, planners, school administrators, and teachers to make school ground environments more attractive and stimulating for PA among school children.

In conclusion, this study described a constructed schoolyard and a natural forest that provided large spaces (a total area of 95 m^2^ per child) and multifunctional school grounds, giving the children several affordances for being physically active during the school day. While our study was not directly linked to the coronavirus disease-2019 (COVID-19) pandemic, the findings may have some implications in the physical and mental health of schoolchildren during the current pandemic. These findings can contribute to inform policies and programs to use school ground environments more effectively, not only to reduce the transmission of infection but also to enhance the physical and mental health of school children through healthy levels of PA as the pandemic continues to be unabated.

## Data Availability Statement

The raw data supporting the conclusions of this article will be made available by the authors, without undue reservation.

## Ethics Statement

The studies involving human participants were reviewed and approved by Norwegian Center for Research Data. Written informed consent to participate in this study was provided by the participants' legal guardian/next of kin.

## Author Contributions

NW has been responsible for the statistical analyses. All the authors have contributed equally to the manuscript.

## Funding

University of South-Eastern Norway—responsible for Open access publication. Part of Open access agreement for University networks.

## Conflict of Interest

The authors declare that the research was conducted in the absence of any commercial or financial relationships that could be construed as a potential conflict of interest.

## Publisher's Note

All claims expressed in this article are solely those of the authors and do not necessarily represent those of their affiliated organizations, or those of the publisher, the editors and the reviewers. Any product that may be evaluated in this article, or claim that may be made by its manufacturer, is not guaranteed or endorsed by the publisher.
